# Clinical and Echocardiographic Factors Influencing Patent Ductus Arteriosus Treatment in Preterm Neonates

**DOI:** 10.3390/children12070936

**Published:** 2025-07-16

**Authors:** Mi Ae Chu, So Young Shin, Jae Hyun Park, Hee Joung Choi

**Affiliations:** 1 Department of Pediatrics, Kyungpook National University Children’s Hospital, Daegu 41404, Republic of Korea; quiremi78@gmail.com; 2 Department of Pediatrics, Keimyung University Dongsan Hospital, Daegu 42601, Republic of Korea; botanic-garden@daum.net (S.Y.S.);

**Keywords:** clinical parameters, echocardiographic parameters, patent ductus arteriosus, preterm neonates, hemodynamic stability

## Abstract

**Objective**: We evaluated how pre-treatment clinical and echocardiographic findings influence treatment decisions for patent ductus arteriosus (PDA) in preterm neonates. **Study Design**: Preterm neonates weighing < 1500 g and diagnosed with PDA were enrolled. They were categorized into conservative, medical, and surgical groups based on treatment. **Results**: A total of 242 preterm neonates (120 boys and 122 girls) participated, with a mean gestational age of 27.9 ± 2.2 weeks and a birth weight of 1034.3 ± 239.3 g. Multivariate logistic regression revealed that oliguria (*p* < 0.001), inotropic drug use (*p* = 0.049), low PDA flow velocity (*p* = 0.039), and left atrial enlargement (*p* = 0.002) were significantly associated with medical or surgical treatment decisions. Additionally, a low base deficit prior to medical therapy was associated with the decision to proceed with surgical intervention after medical treatment failure (*p* = 0.006). **Conclusions**: Oliguria, inotropic drug use, low PDA flow velocity, and left atrial enlargement were significantly associated with aggressive treatment decisions in preterm neonates with PDA. Furthermore, a low base deficit influenced the need for surgery following medical therapy failure. Our findings suggest that comprehensive monitoring of both clinical and echocardiographic factors may support treatment decision-making in PDA management in preterm neonates.

## 1. Introduction

In premature infants, the incidence of patent ductus arteriosus (PDA) ranges from 70–80% of those born at 25–28 weeks of gestation and up to 90% of those born at 24 weeks [1,2]. The hemodynamic consequences of PDA in preterm infants lead to pulmonary overcirculation and systemic hypoperfusion, which contribute to significant morbidities, including chronic lung disease, intraventricular hemorrhage, necrotizing enterocolitis, retinopathy of prematurity, and an elevated risk of mortality [2,3]. Consequently, timely and appropriate management of PDA is critical for improving neonatal outcomes.

Management of PDA in preterm infants remains a contentious issue among neonatologists, with a wide range of pharmacotherapeutic strategies available, spanning from conservative to more aggressive approaches. Current trends in clinical practice favor a selective management approach that aims to balance the potential benefits and risks for each individual patient [4,5,6]. Although definitive treatment guidelines for PDA are still lacking, optimizing management through a combination of comprehensive clinical evaluation, echocardiography, and, potentially, biomarkers monitored serially is essential to improving outcomes [4,7].

McNamara and Sehgal suggested a widely accepted staging system for hemodynamically significant PDA (HS PDA) to guide treatment decisions [8]. However, its consistent and uniform application remains challenging in clinical settings. Several studies have highlighted various factors influencing the spontaneous closure of PDA, the necessity for intervention, and the efficacy of pharmacological treatments, with heterogeneous results in preterm neonates [3,9]. Nonetheless, few studies have concurrently evaluated both echocardiographic and clinical parameters at the time of treatment to predict the most appropriate intervention for very preterm infants.

This study aimed to assess how pre-treatment clinical and echocardiographic findings influence the decision-making process in PDA management. The novelty of this study lies in its comprehensive evaluation of both clinical and echocardiographic parameters obtained at the actual time of treatment decision-making, rather than at predetermined time points. Specifically, we investigated the differences in these parameters across various treatment strategies and identified the key determinants influencing decisions for medical or surgical intervention. Furthermore, we explored the factors that influence the decision to proceed with surgical treatment after medical therapy failure.

## 2. Materials and Methods

### 2.1. Patient Population

This retrospective study included preterm neonates born at less than 37 weeks of gestation with birth weights below 1500 g who were admitted to the neonatal intensive care unit at Keimyung University Dongsan Hospital, Korea, between 2014 and 2018. Neonates diagnosed with PDA by 2D echocardiography were enrolled in the study. Exclusion criteria included neonates with birth weights less than 500 g, those with complex congenital heart diseases or other major anomalies, neonates receiving prophylactic PDA treatment, and those who died within the first three postnatal days. Additionally, patients with persistent pulmonary hypertension treated with inhaled nitric oxide and those with early-onset sepsis were excluded due to their potential impact on hemodynamic stability.

Patients were categorized into three groups based on the method of PDA treatment: conservative, medical, or surgical. The treatment decision was made at the discretion of the attending clinician based on clinical findings and echocardiographic results. Conservative treatment strategies included maintaining blood pressure with inotropic agents or hydrocortisone, restricting fluid intake, and providing respiratory support. Oral ibuprofen was administered for three consecutive days as part of the medical treatment regimen. Surgical ligation was performed in patients who demonstrated poor responses to both medical and conservative management; those who were contraindicated to medical therapy due to conditions such as thrombocytopenia (platelet count < 100,000/mm^3^), bleeding tendencies, azotemia (serum creatinine > 1.0 mg/dL), or oliguria; or those displaying evidence of necrotizing enterocolitis.

This study was approved by the Institutional Review Board of Keimyung University Dongsan Hospital (approval number: DSMC 2020-03-057).

### 2.2. Clinical and Echocardiographic Data

Demographic data, including sex, birth weight, and gestational age, were collected for all patients. To investigate factors influencing treatment decisions, we analyzed the following clinical and echocardiographic parameters: (1) Clinical parameters included the mode of ventilator support (non-invasive or invasive); mean airway pressure; oxygen concentration; serum creatinine level; serum pH; base deficit; and the incidence of apnea, bradycardia (defined as a heart rate < 90 beats per minute), oliguria, and inotropic drugs use. (2) Echocardiographic parameters included the size of the PDA, measured as the pulmonary end diameter, as well as flow velocity and the left atrium-to-aortic root (LA/Ao) ratio. Other echocardiographic factors assessed were the size of mitral and tricuspid valve annuli; inflow velocities of mitral and tricuspid valves; and the incidence of left atrial (LA) enlargement, mitral regurgitation, and atrial septal defects (ASDs).

Echocardiographic examinations were conducted by a single pediatric cardiologist using a Simens Acuson Sequoia ultrasound scanner with a 10 MHz transducer (Siemens Medical Solutions, Mountain View, CA, USA). All enrolled patients underwent their first 2D echocardiographic examination within one week after birth, and follow-up echocardiographic examinations were conducted on an individualized basis according to clinical indications. Clinical and echocardiographic data for the conservative group were collected between 4 and 14 days postnatally, while data for the medical and surgical groups were collected immediately before the administration of medical treatment or the performance of ductal ligation.

### 2.3. Statistical Analysis

Data are presented as frequencies and means ± standard deviations. The chi-squared test was used to analyze categorical data, while an unpaired *t*-test was used to compare continuous data between the two groups. Multivariate logistic regression analysis, adjusted for gestational age, was conducted to assess factors influencing PDA treatment, and the results are presented as Exp(B) with 95% confidence intervals. Statistical analyses were performed using SPSS Statistics (version 21.0; IBM Corp., Armonk, NY, USA), with a *p*-value < 0.05 considered statistically significant.

## 3. Results

### 3.1. Patient Characteristics

**Figure 1:**
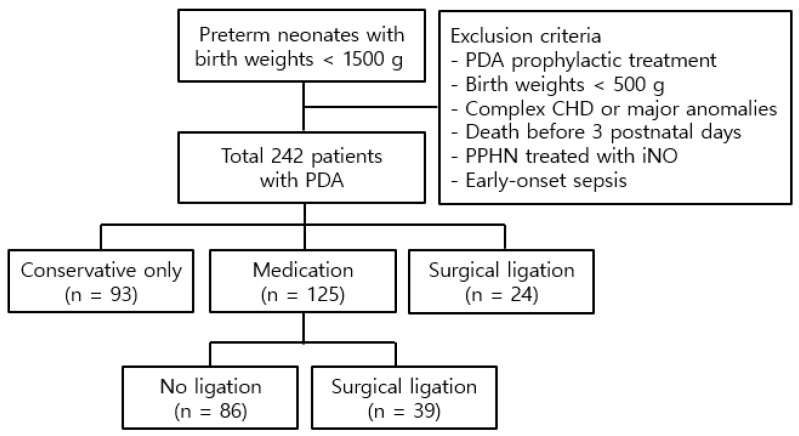
Study population. Abbreviations: PDA, patent ductus arteriosus; CHD, congenital heart disease; PPHN, persistent pulmonary hypertension of the newborn; iNO, inhaled nitric oxide.

A total of 242 preterm neonates diagnosed with PDA were enrolled, consisting of 120 boys and 122 girls, with a mean gestational age and birth weight of 27.9 ± 2.2 weeks and 1034.3 ± 239.3 g, respectively (Figure 1). Among these patients, 93 received conservative treatment, while 149 underwent aggressive treatment, including medical intervention in 125 patients and surgical intervention in 24 patients. Of the 125 patients treated medically, 39 required subsequent surgery due to an inadequate response to pharmacotherapy. Medical treatment was initiated at 15.1 ± 4.7 days postnatally, while surgical treatment was performed at 17.9 ± 10.6 days postnatally. Patients requiring surgery after failed pharmacotherapy underwent the procedure at 18.0 ± 8.8 days postnatally.

### 3.2. Clinical and Echocardiographic Parameters Related to PDA Treatment

Patients who underwent aggressive treatment (medical or surgical) had a higher incidence of apnea (*p* = 0.001), oliguria (*p* < 0.001), and inotropic drug use (*p* = 0.004) and higher serum creatinine levels (*p* = 0.046) in clinical parameters compared to those receiving conservative treatment (Table 1). They also had a larger PDA size (*p* < 0.001), lower PDA flow velocity (*p* = 0.010), a higher LA/Ao ratio (*p* < 0.001) and MV inflow velocity (*p* = 0.009), and a higher incidence of LA enlargement (*p* <0.001) and ASDs (*p* < 0.001) in echocardiographic parameters compared to those with conservative treatment.

Multivariate logistic regression analysis identified oliguria (*p* < 0.001, Exp(B) = 3.229), inotropic drug use (*p* = 0.049, Exp(B) = 3.230), low PDA flow velocity (*p* = 0.039, Exp(B) = 0.175), and LA enlargement (*p* = 0.002, Exp(B) = 7.652) as significant factors influencing medical or surgical treatment decisions (Table 2).

When comparing the medical and surgical treatment groups with the conservative treatment group (Table 1), patients who received medical treatment had a higher incidence of apnea and oliguria, a larger PDA size, lower PDA flow velocity, a higher LA/Ao ratio and MV inflow velocity, and a higher incidence of LA enlargement and ASDs. In addition, patients who underwent surgical treatment showed a higher incidence of bradycardia, oliguria, and inotropic drug use; higher levels of serum creatinine; a lower pH and base deficit; a larger PDA size; lower PDA flow velocity; a higher LA/Ao ratio and MV inflow velocity; and a higher incidence of LA enlargement and ASDs compared to those who underwent conservative treatment.

Multivariate logistic regression analysis revealed that inotropic drug use (*p* = 0.045, Exp(B) = 3.817), low PDA flow velocity (*p* = 0.028, Exp(B) = 0.091), and LA enlargement (*p* = 0.025, Exp(B) = 4.569) significantly influenced the decision to undergo medical treatment (Table 2). Meanwhile, oliguria (*p* = 0.036, Exp(B) = 9.622) and inotropic drug use (*p* < 0.001, Exp(B) = 4.565) were significantly associated with the decision to proceed with surgical treatment despite initial conservative management.

### 3.3. Clinical and Echocardiographic Parameters Related to Medical Treatment Failure

Patients who required surgery following medical treatments had lower pH and base deficit levels compared to those who did not require surgery (Table 3). The multivariate logistic regression analysis identified a low base deficit (*p* = 0.006, Exp(B) = 0.838) as a significant indicator of medical treatment failure (Table 2).

For patients who received both medical and surgical treatments, differences between pre-medical and pre-surgical conditions were examined. While there was an increase in PDA flow velocity before surgery compared to before medical treatment, the need for oxygen and inotropic drugs, as well as serum creatinine levels, significantly increased before surgical intervention.

## 4. Discussion

In this study, oliguria, inotropic drug use, low PDA flow velocity, and LA enlargement were significantly associated with medical and surgical treatment decisions in preterm neonates with PDA, indicating the failure of conservative management. Additionally, a low base deficit prior to medical treatment was associated with an increased likelihood of surgical intervention due to medical therapy failure.

The management of PDA in preterm infants requires critical decisions regarding whether the ductus should be treated, the appropriate method of treatment (medical or surgical), and the optimal timing of intervention. In making these decisions, the definition of HS PDA proposed by McNamara and Sehgal is commonly used. This definition incorporates both clinical and echocardiographic parameters to determine the best treatment approach for PDA [8].

Among echocardiographic parameters, PDA size and flow velocity are the most simply measurable indicators. Some studies suggest that a PDA width < 2.5 mm on the third day of life or a maximum shunt velocity > 1.165 m/s 48 h after birth predicts spontaneous closure [3]. However, other studies indicate that PDA diameter or velocity alone may not reliably indicate closure [10,11,12]. To improve accuracy, factors such as PDA size adjusted for body weight or the PDA/left pulmonary artery ratio have been proposed [11,12]. A PDA size/weight > 1.5 mm/kg within 12–48 hours after birth and a PDA size/weight ≥ 2.0 mm/kg at 72 hours correlate with the need for surgical intervention or other treatments [13,14]. Moreover, incorporating the LA/Ao ratio improves predictive accuracy, with combined indicators of PDA size/weight > 3.2 mm/kg and LA/Ao > 1.4 at a 72 h postnatal age, predicting the need for intervention in preterm infants with a gestational age ≤ 30 weeks [9]. In our study, low PDA flow velocity and LA enlargement were significant determinants in treatment decisions, while PDA diameter was not a significant factor. This may be because PDA diameter and LA/Ao ratio measurements are influenced by the angle and resolution of echocardiographic imaging, whereas velocity measurements are less affected by infant weight or gestational age, thereby minimizing measurement inaccuracies. Although assessments of LA enlargement can be subjective, they may reflect an infant’s hemodynamic status better.

To compensate for the uncertainty of these echo measurements, it is essential to incorporate clinical findings in the decision-making process. In our study, oliguria and inotropic drug use were significant determinants of treatment decisions. Specifically, oliguria was significantly more frequent in both the medical and surgical treatment groups compared to the conservative treatment group. Similarly, Wu et al. identified surfactant administration and dopamine use as predictors of surgical intervention in neonates with a very low birth weight [15]. Although limited research exists on whether oliguria prior to treatment directly influences aggressive PDA management decisions, studies have examined urine output following pharmacotherapy as a marker of treatment response. However, the results across studies have been inconsistent. One study reported no significant differences in urine output between successful and unsuccessful indomethacin treatment, whereas another found that higher urine output during medication was associated with complete PDA closure [16]. Conversely, a separate study suggested that a decrease in urine output following medication correlated with successful PDA closure [17].

Echocardiographic parameters linked to medical treatment response include a small PDA size (≤2 mm) and a high maximum velocity (>180 cm/s), which have been identified as strong predictors of successful PDA closure following pharmacotherapy [18,19,20]. Additionally, non-responders to acetaminophen treatment often exhibit high preload echocardiographic parameters, such as increased left ventricular output, right ventricular output, and LA/Ao ratios [21].

Although our study did not identify specific echocardiographic indicators of treatment failure, we observed that patients with a low base deficit prior to treatment initiation were at an increased risk of medical therapy failure. Studies on pre-treatment metabolic acidosis have indicated that lower pH values (mean arterial pH ≤ 7.26) within the first 48 h predict the subsequent need for surgical ligation [22]. Additionally, higher base deficit and hematocrit levels may independently predict successful indomethacin treatment in very low birth weight infants with PDA, with recommendations to initiate treatment before the base deficit drops below −4.56 [20]. Therefore, managing metabolic acidosis before treatment may be beneficial.

Our study also revealed unique and less commonly reported differences between pre-medical and pre-surgical treatments in PDA management. Following pharmacotherapy, while echocardiographic parameters such as PDA size and flow velocity either remained stable or showed improvements, clinical parameters worsened, as evidenced by elevated serum creatinine levels and increased reliance on oxygen and inotropic drugs. This clinical deterioration often necessitated surgical intervention.

The strength of this study lies in its unique approach in analyzing both clinical and echocardiographic parameters at the time of medical or surgical treatment decisions. Previous studies have typically relied on echocardiographic findings obtained at 12–48 or 72 h after birth to predict the need for treatment. However, given that echocardiographic findings can change rapidly, assessing them when clinical symptoms are observed and treatment decisions are made offers more valuable insights. By integrating both echocardiographic and clinical findings, our study offers a more comprehensive understanding of HS PDA.

Several limitations of this study must be considered. First, as a single-center, retrospective observational study, there is potential for statistical bias, especially due to the small sample size in certain subgroups. Second, perinatal factors influencing PDA outcomes were not considered. Third, the scope of our echocardiographic analysis was limited by the retrospective nature of the study and the reliance on available ultrasound data from routine clinical practice.

## 5. Conclusions

In preterm neonates with PDA, oliguria, inotropic drug use, low PDA flow velocity, and LA enlargement were significantly associated with medical or surgical treatment decisions, potentially reflecting the clinical judgment of hemodynamic significance. Additionally, a low base deficit prior to medical treatment influenced the subsequent surgical intervention following medical therapy failure. However, no single clinical or echocardiographic parameter consistently predicted PDA-related outcomes. Therefore, close monitoring of clinical signs indicating hemodynamic compromise and frequent bedside echocardiographic examinations are crucial for the early identification and targeted treatment of PDA in preterm neonates.

## Figures and Tables

**Table 1:** Clinical and echocardiographic parameters related to PDA treatment.

	Conservative (*n* = 93)	Intervention (*n* = 149)	Medical (*n* = 125)	Surgical (*n* = 24)	*p*-Value
Mean airway pressure (cmH_2_O)	9.5 ± 2.6	9.0 ± 3.1	9.0 ± 2.7	9.1 ± 4.6	0.542
FiO_2_	0.23 ± 0.05	0.22 ± 0.05	0.22 ± 0.05	0.22 ± 0.06	0.731
Oxygen use	14.6%	13.8%	12.4%	20.8%	0.861
Apnea	15.2%	34.9%	37.6% *	20.8%	0.001
Bradycardia	6.5%	14.2%	10.2%	20.8% ^†^	0.092
Oliguria	18.7%	51.0%	46.4% *	75.0% ^†^	<0.001
Inotropic drug use	27.2%	45.6%	38.4%	83.3% ^†^	0.004
Mechanical ventilator support					0.067
No	9.8%	9.9%	4.8%	4.2%	
Non-invasive	67.4%	60.4%	61.6%	54.2%	
Invasive	22.8%	34.9%	33.6%	41.7%	
Creatinine (mg/dL)	0.88 ± 0.51	1.06 ± 0.71	0.92 ± 0.38	1.87 ± 1.40 ^†^	0.046
pH	7.25 ± 0.13	7.24 ± 0.10	7.25 ± 0.09	7.19 ± 0.12 ^†^	0.273
Base deficit (mmol/L)	−9.81 ± 5.75	−10.45 ± 5.35	−10.00 ± 4.95	−12.79 ± 6.76 ^†^	0.304
PDA size (mm)	1.76 ± 0.55	2.17 ± 0.61	2.14 ± 0.57 *	2.33 ± 0.79 ^†^	<0.001
PDA velocity (m/s)	1.99 ± 0.77	1.74 ± 0.57	1.75 ± 0.58 *	1.72 ± 0.50 ^†^	0.010
LA/Ao ratio	1.39 ± 0.31	1.59 ± 0.30	1.45 ± 0.30 *	1.70 ± 0.28 ^†^	<0.001
Mitral valve inflow (m/s)	0.60 ± 0.17	0.70 ± 0.17	0.69 ± 0.16 *	0.73 ± 0.21 ^†^	0.009
Tricuspid valve inflow (m/s)	0.65 ± 0.15	0.66 ± 0.13	0.66 ± 0.13	0.68 ± 0.14	0.590
LA enlargement	5.6%	37.1%	31.2% *	65.2% ^†^	<0.001
Mitral regurgitation	16.7%	16.9%	17.7%	13.0%	0.976
Atrial septal defect	74.2%	95.9%	95.2% *	100.0% ^†^	<0.001

The data are presented as the number (%) and mean ± SD. chi-squared and unpaired t-tests were used for categorical and continuous variables, respectively. * *p* < 0.05: comparison of parameters between the conservative group and the medical group. ^†^ *p* < 0.05: comparison of parameters between the conservative group and the surgical group. Abbreviations: PDA, patent ductus arteriosus; FiO_2_, fraction of inspired oxygen; LA/Ao, left atrium-to-aortic root; LA, left atrium.

**Table 2:** Multivariate logistic regression analysis of factors influencing treatment failure.

		Exp(B)	95% CI	*p*-Value
**Conservative treatment failure**				
Intervention vs. Conservative treatment	Oliguria	3.229	1.709–6.100	<0.001
	Inotropic drug use	3.230	1.007–10.354	0.049
	PDA velocity	0.175	0.033–0.918	0.039
	LA enlargement	7.652	2.117–27.655	0.002
Medical vs. Conservative treatment	Inotropic drug use	3.817	1.048–44.354	0.045
	PDA velocity	0.091	0.011–0.777	0.028
	LA enlargement	4.569	1.212–17.216	0.025
Surgical vs. Conservative treatment	Oliguria	9.622	1.163–79.590	0.036
	Inotropic drug use	4.565	2.110–9.879	<0.001
**Medical treatment failure**				
Medical treatment only vs. Surgical treatment after medical treatment	Base deficit	0.838	0.721–0.970	0.006

Abbreviations: CI, confidence interval; PDA, patent ductus arteriosus; LA, left atrial.

**Table 3:** Clinical and echocardiographic parameters related to medical treatment failure.

	Medical Treatment Only (*n* = 86)	Surgical Treatment After Medical Treatment (*n* = 39)	
	Before Medication	Before Medication	Before Surgery
Mean airway pressure (cmH_2_O)	9.1 ± 2.6	8.8 ± 2.9	9.3 ± 3.1
FiO_2_	0.22 ± 0.05	0.22 ± 0.05	0.24 ± 0.04
Oxygen use	13.4%	10.3%	35.1% ^†^
Apnea	32.6%	48.7%	38.5%
Bradycardia	14.1%	10.3%	15.4%
Oliguria	48.8%	41.0%	43.6%
Inotropic drug use	34.9%	46.2%	79.5% ^†^
Mechanical ventilator support			
No	4.1%	1.9%	2.6%
Non-invasive	53.0%	59.0%	41.0%
Invasive	31.4%	38.5%	56.4%
Creatinine (mg/dL)	0.88 ± 0.40	1.00 ± 0.30	1.56 ± 1.36 ^†^
pH	7.26 ± 0.09	7.22 ± 0.08 *	7.20 ± 0.12
Base deficit (mmol/L)	−9.20 ± 5.19	−11.76 ± 3.86 *	−12.59 ± 6.28
PDA size (mm)	2.14 ± 0.59	2.14 ± 0.52	2.22 ± 0.55
PDA velocity (m/s)	1.74 ± 0.63	1.76 ± 0.45	2.02 ± 0.46 ^†^
LA/Ao ratio	1.60 ± 0.30	1.50 ± 0.26	1.52 ± 0.36
Mitral valve inflow (m/s)	0.66 ± 0.15	0.77 ± 0.15	0.85 ± 0.22
Tricuspid valve inflow (m/s)	0.65 ± 0.15	0.67 ± 0.10	0.73 ± 0.15
LA enlargement	29.7%	34.3%	26.1%
Mitral regurgitation	19.2%	14.3%	9.1%
Atrial septal defect	96.5%	92.3%	94.7%

The data are presented as the number (%) and mean ± standard deviation. * *p* < 0.05: comparison of parameters before medication, between medications only, and ligation after medication. ^†^ *p* < 0.05: comparison of parameters before medication versus before ligation in patients with ligation after medication. Abbreviations: FiO_2_, fraction of inspired oxygen; PDA, patent ductus arteriosus; LA/Ao, left atrium-to-aortic root; LA, left atrium.

## Data Availability

The original contributions presented in this study are included in the article. Further inquiries can be directed to the corresponding author.

## Abbreviations

The following abbreviations are used in this manuscript:

PDA	Patent ductus arteriosus
HS PDA	Hemodynamically significant PDA
LA/Ao	Left atrium-to-aortic root
LA	Left atrium
ASDs	Atrial septal defects
